# The state of medical oncology in Senegal

**DOI:** 10.3332/ecancer.2019.941

**Published:** 2019-07-25

**Authors:** Mamadou Diop

**Affiliations:** Cancer Institute of Cheikh Anta Diop University, Hôpital Aristide Le Dantec, 30 Ave Pasteur, Dakar 15674, Fann, Senegal

**Keywords:** oncology, Africa, Senegal

## Abstract

Senegal has only one medical oncology specialist, who works at the Cancer Institute, but chemotherapy is performed in several public and private sites in the capital city of Dakar by competent general practitioners and oncology surgeons. There are several key needs. Human resources development is necessary that includes an offer of fellowships for full specialisation for freshly graduated doctors, combined with capacity training of experienced general practitioners, without forgetting to add chemotherapy in the training curricula of nurses. There is a need for a substantial subsidy of anti-cancer drugs, especially targeted treatments, and their control by the national drug-control laboratory. All this requires the centralised management of anticancer drugs with a technical platform adapted for their secure storage, preparation and delivery.

## Background

**Figure 1.**
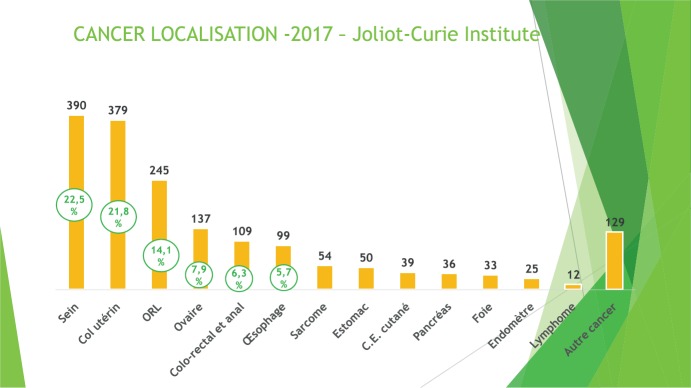
Cancer localisation, Cancer Institute tumour registry, 2017 (unpublished data).

The incidence of cancer in Senegal is estimated at 6,646 new cases per year with a mortality rate of about 72%, and with breast cancer and cervical cancer being the predominant locations (Figure 1) [1]. The high mortality is mainly due to late consultation, a lack of human resources and adequate equipment.

**Figure 2.**
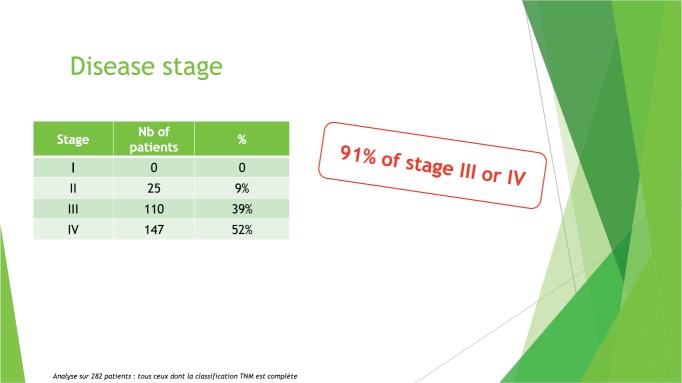
Disease stage, Cancer Institute tumour registry, 2017 (unpublished data).

According to the 2017 cancer registry of the Joliot-Curie Institute (Cancer Department of A Le Dantec Hospital), which is the only cancer centre in Senegal, more than 90% of cancer patients arrive at stages III and IV, when care and treatment often include chemotherapy (Figure 2).

The demand for chemotherapy is very high, consisting of 75% neoadjuvant chemotherapy for all cancers despite overuse of primary surgery for sites, such as ‘head and neck’, ‘lung’ and ‘digestive’ [2–5].

**Figure 3.**
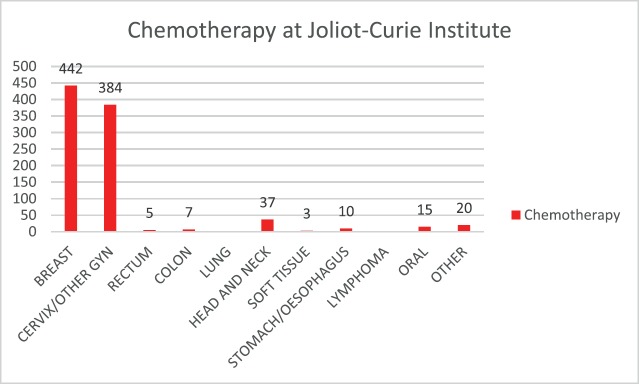
Distribution of chemotherapy sites (unpublished data).

Of the approximately 1,612 therapeutic acts carried out at the Joliot-Curie Institute, 923 (almost 60%) are chemotherapy acts (Figure 3).

## Insufficient human resources

Senegal has only one medical oncology specialist, who works at the Cancer Institute, but chemotherapy is performed in several public and private sites in the capital city of Dakar by competent general practitioners and oncology surgeons. Three nurses from the Cancer Institute have completed 6 months of practical training at the Institute Paoli Calmettes in Marseille, France and are now acting as training instructors for services that want to open day-care chemotherapy. In this way, chemotherapy has been carried out for a year at the regional hospital of Thiès (70 km from Dakar), which is the only known structure outside the capital where chemotherapy is performed. Two doctors are finishing their specialisation in Morocco and Ivory Coast, with a training cost of 30,000 euros/year, for a total duration of 4 years.

The Human Resources Development strategic axis of the 2016–2020 National Strategic Plan for the Fight Against Cancer has planned to have 50% of doctors and general surgeons follow seminars and practical internships for chemotherapy training, in order to achieve a 30% 5-year survival rate for treated cancer patients. There is also an annual offer of three fellowships for full specialisation in medical oncology.

## The inadequate technical platform

In general, after the doctor prescribes chemotherapy, it is the patient who finds the means of obtaining the anti-cancer drugs: either at the public hospital through the National Pharmacy Supply or at private pharmacies. The storage, preparation and delivery of chemotherapy are not carried out in a secure way in a centralised reconstitution unit.

**Figure 4.**
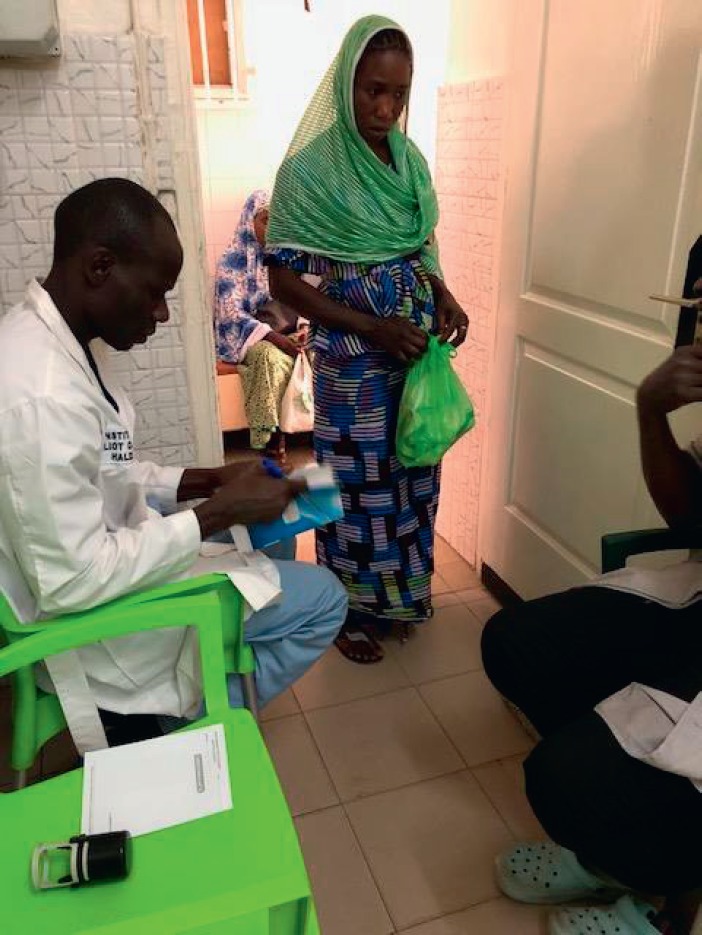
A patient presenting at her chemotherapy appointment with her medications.

**Figure 5.**
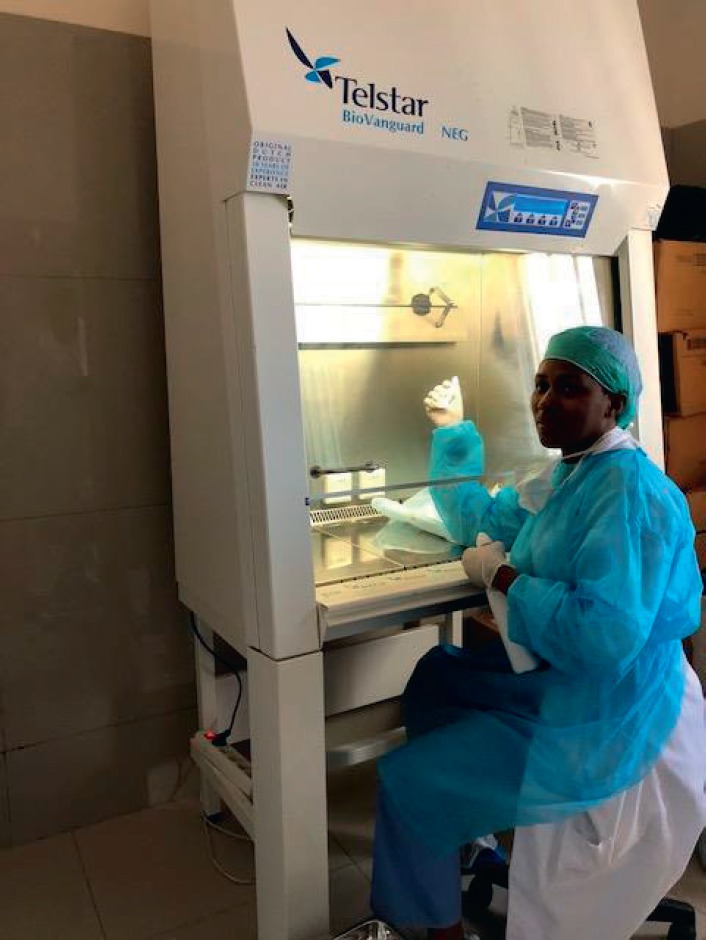
A single hood for the preparation of anticancer drugs.

Patients bring their medication in plastic bags (Figure 4). Only one functional hood exists for the safety of the nursing staff of the Institute (Figure 5), but it is not used systematically because of the delays it adds to patient care. There are no special chemotherapy waste-management measures different from the usual measures.

In paediatric oncology, with the programme of the Franco-African Group of Paediatric Oncology, chemotherapy is free and drugs are provided and available in a controlled and secure manner.

Implantable chambers are mainly used in private structures because of their cost and their inaccessibility. Two hundred euros are required for the purchase and implementation.

## High overall cost of cancer care

The high overall cost of managing cancer (between 950,000 CFA (1,835 USD) and 2,250,000 CFA (4345 USD)), as well as the influence that people’s beliefs have on the choice of therapeutic options, explain why the majority of cancer patients start or finish their care in traditional medicine. This phenomenon results in an increase in mortality due to late diagnosis, and also in a failure of treatment compliance.

Anti-cancer drugs, some of which are very expensive (more than 1 million CFA francs for a cure with a minimum of four courses), must be subsidised, as are anti-retroviral AIDS drugs and anti-tuberculosis chemotherapy. Antineoplastic drugs not covered by the Universal Health Coverage policy are at the total expense of the patients for a total average cost of the treatment protocol of approximately 1,500 euros per patient.

In the 2016–2020 national strategic plan, with a budget of 73 million euros, 89% of which is devoted to diagnostic and therapeutic management, it is planned to formally subsidise targeted treatments to make them accessible. For the moment, including anti-cancer drugs on the list of essential medicines with calls for tenders has allowed an influx of generic medication, and a fall in the costs of conventional and essential anticancer drugs, such as anthracyclines by 70%.

A grant of 1.5 million euros per year was recently announced by the government on the basis of a technical and financial proposal which will be renewed every year.

Non-governmental organisations, such as Direct Relief based in the United States of America, retrieve and send us medicines before the expiry date. We have already received 500 boxes of xeloda, 1,500 boxes of filgrastim and 1,000 boxes of granisetron. These drugs are redistributed free of charge to our patients as needed.

## Conclusion

In summary, our needs in medical oncology are three-fold:

- A human resources development that includes an offer of fellowships for full specialisation for freshly graduated doctors, combined with capacity training of experienced general practitioners without forgetting to add chemotherapy in the training curricula of nurses.
- A substantial subsidy of anti-cancer drugs, especially targeted treatments, and their control by the national drug-control laboratory.
- Centralised management of anticancer drugs with a technical platform adapted for their secure storage, preparation and delivery.

## Conflicts of interest

The author has no conflicts of interest to declare.

## Funding

The author did not receive any funding for this work.
